# Contrast Enhancement Ultrasound Improves Diagnostic Accuracy for Thyroid Nodules: A Prospective Multicenter Study

**DOI:** 10.1210/jendso/bvad145

**Published:** 2023-11-28

**Authors:** Jianming Li, Jianping Dou, Huarong Li, Fan Xiao, Jie Yu, Mingxing Xie, Ping Zhou, Lei Liang, Guiming Zhou, Ying Che, Cun Liu, Zhibin Cong, Fangyi Liu, Zhiyu Han, Ping Liang

**Affiliations:** Department of Interventional Ultrasound, Fifth Medical Center of Chinese PLA General Hospital, Beijing 100853, China; Department of Interventional Ultrasound, Fifth Medical Center of Chinese PLA General Hospital, Beijing 100853, China; Department of Ultrasound, Aero-space Center Hospital, Beijing 100049, China; Department of Interventional Ultrasound, Fifth Medical Center of Chinese PLA General Hospital, Beijing 100853, China; Department of Interventional Ultrasound, Fifth Medical Center of Chinese PLA General Hospital, Beijing 100853, China; Department of Ultrasound Medicine, Union Hospital, Tongji Medical College, Huazhong University of Science and Technology, Wuhan 430022, China; Department of Ultrasound, Third Xiangya Hospital, Central South University, Guangdong 410008, China; Department of Ultrasound, Aero-space Center Hospital, Beijing 100049, China; Department of Ultrasound, Tianjin Medical University General Hospital, Tianjin 300052, China; Department of Ultrasound, The First Affiliated Hospital of Dalian Medical University, Dalian 116011, China; Department of Ultrasound, Jinan Central Hospital, Shandong First Medical University & Shandong Academy of Medical Sciences, Jinan 250199, China; Department of Ultrasound, Affiliated Hospital of Changchun University of Chinese Medicine, Changchun 130021, China; Department of Interventional Ultrasound, Fifth Medical Center of Chinese PLA General Hospital, Beijing 100853, China; Department of Interventional Ultrasound, Fifth Medical Center of Chinese PLA General Hospital, Beijing 100853, China; Department of Interventional Ultrasound, Fifth Medical Center of Chinese PLA General Hospital, Beijing 100853, China

**Keywords:** thyroid nodule, thyroid carcinoma, ultrasound, contrast-enhanced ultrasound

## Abstract

**Objective:**

To evaluate potential improvements in the diagnosis of thyroid nodules when conventional ultrasound (US) is combined with contrast-enhanced US (CEUS).

**Methods:**

We recruited 515 participants with 323 malignant and 192 benign nodules, who underwent both US and CEUS examinations at 8 different medical centers in China between October 2020 and October 2021. We assessed the malignancy of thyroid nodules in US using the American College of Radiology (ACR) Thyroid Imaging Reporting and Data System (TIRADS). Diagnostic criteria for US and US + CEUS were developed by investigators based on evaluations of sonographic features. Using multivariate logistic regression and receiver operating characteristic (ROC) analysis, we compared diagnostic performance between the 2 methods based on criteria identified by investigators and via statistical models.

**Results:**

On the basis of diagnostic criteria identified by investigators, we measured statistically significant differences in area under the curve (AUC) values between ACR TIRADS (0.83) and CEUS TIRADS (0.87; *P* < .001). On the basis of diagnostic regression models, we found statistically significant differences in AUC values between US (0.76) and US + CEUS (0.84; *P* = .001). Models based on US + CEUS outperformed those based on US alone (Akaike information criterion of 347.7 and significant improvement in integrated discrimination). These results were confirmed by similar analyses applied to a validation cohort.

**Conclusion:**

The accuracy of conventional US for differentiating between benign and malignant thyroid nodules can be improved by combining this approach with CEUS.

The global incidence of thyroid nodules has increased substantially during the past 3 decades, partly as a consequence of the widespread use of ultrasound (US) for diagnosis [1]. Reliable diagnosis of malignant and benign nodules is critical for prognostic purposes and to avoid overdiagnosis, overtreatment, and overuse of medical resources. The American College of Radiology Thyroid Imaging Reporting and Data System (ACR TIRADS) is a risk stratification system that provides standardized guidelines for US-based diagnosis and management of thyroid nodules [2, 3].

A prior study has shown that when compared or integrated with gray-scale US features, vascular information by using Doppler/Power Doppler did not provide any added benefit for predicting thyroid malignancy [4]. Following this finding, vascular characteristics were no longer used to classify thyroid nodules in clinical practice, and some scholars have questioned whether vascular information from contrast media contributes to US-based diagnostic performance.

Contrast-enhanced ultrasound (CEUS), a modality that is currently adopted in clinical practice, is primarily used for the evaluation of liver lesions, showing substantial clinical value for this purpose [5]. Some practitioners have adopted CEUS for visualizing unique microvascularization via microbubbles and have provided suggestive evidence that CEUS may improve the diagnosis of thyroid cancer compared with conventional US [6-10]. Notwithstanding the promising results of these retrospective studies, guidelines from the European Federation of Societies for Ultrasound in Medicine and Biology (EFSUMB) characterize research into CEUS as still exploratory and do not therefore recommend this methodology for clinical diagnosis of thyroid nodules [11]. This lack of certainty about the efficacy of CEUS probably reflects the paucity of reports that have prospectively evaluated the diagnostic performance of this methodology for thyroid cancer diagnosis.

In this study, we first evaluate diagnostic performance for differentiating between benign and malignant thyroid nodules using conventional US and US combined with CEUS in the prospective multicenter study. Diagnostic performance was quantified using receiver operating characteristic (ROC) analysis and compared across methods with reference to criteria identified by investigators as well as statistical models.

## Materials and Methods

**Figure 1.**
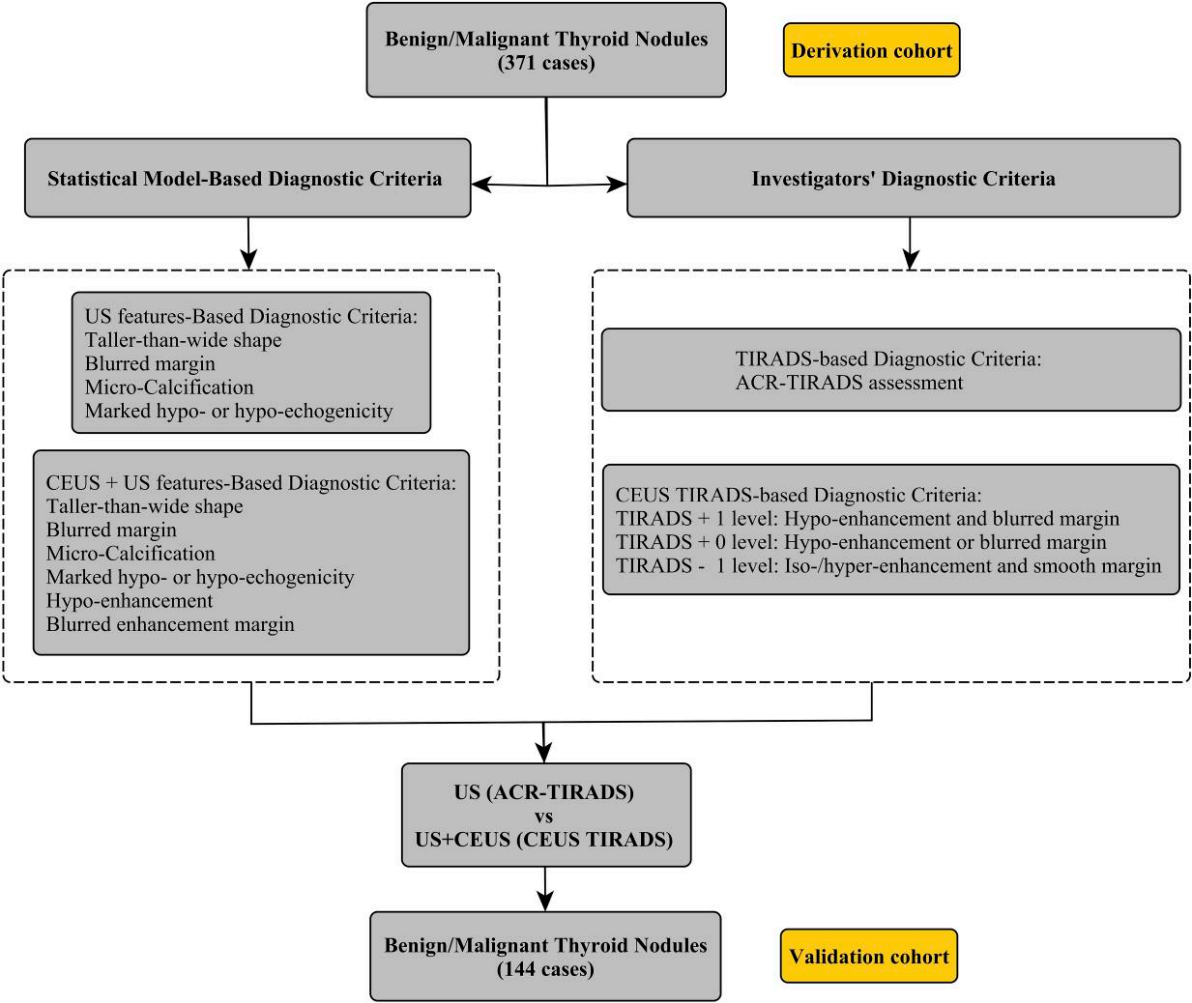
Flowchart of this pilot study.

This prospective multicenter diagnostic study was conducted between October 1, 2020, and October 1, 2021. It was approved by the institutional review board of the 9 participating medical centers, and written informed consent was obtained from all participants. The flowchart in Fig. 1 describes the overall study design, including participant selection, approaches for identifying diagnostic criteria based on statistical models, and the criteria formulated by investigators for differentiating between benign and malignant thyroid nodules. We constructed a derivation cohort from data collected between October 1, 2020, and May 1, 2021, and a validation cohort from data collected between May 2, 2021, and October 1, 2021.

### Participant Selection

We adopted the following inclusion criteria: (i) age ≥18 years; (ii) examination with nonenhanced (conventional) US and CEUS; and (iii) presence of thyroid nodule visible on US that warranted biopsy or surgery. The exclusion criteria were as follows: (i) history of neck surgery; (ii) pregnancy or allergy to contrast agents; (iii) poor image quality due to breathing or nonstandard procedures; and (iv) thyroid nodules without definitive confirmation of benign or malignant pathology.

### Equipment

Both conventional US and CEUS examinations were performed using a GE LOGIQ E9 general imaging system (GE Healthcare) equipped with a high-frequency linear array probe (L 2-9-D) and contrast pulse sequencing. The mechanical index was 0.2, and the focal zone was 10 to 25 mm for CEUS. The Sonazoid contrast agent solution was obtained by mixing 16 μL of dry powder preparation with 5 mL of sterile saline. The contrast agent solution was injected intravenously as a bolus at a dose of 1.2 mL, followed by a flush of 5 mL of 0.9% sodium chloride solution for each CEUS examination.

### Image Acquisition

The US examinations were carried out at the Radiology Departments of participating medical centers. After obtaining conventional US images across longitudinal and transverse sections, 2 mL of the Sonazoid solution was injected into the antecubital vein and then flushed with 5 mL saline solution. The thyroid was scanned across transverse and longitudinal sections from the point of contrast administration to the region of contrast wash-out for a total of 5 minutes. During the first 3 minutes, images were acquired continuously. During the subsequent 2 minutes, image acquisition lasted 5 seconds every 30 seconds. The US and CEUS images were stored in DICOM format on the hard drive of the GE LOGIQ E9 general imaging system.

### Image Evaluation by Radiologists

Eight senior radiologists (with more than 10 years of experience in thyroid US imaging), who were blind to clinical information regarding patients, independently evaluated diagnostic sonographic features from thyroid glands that contained a nodule on both US and CEUS images. They were based at the participating medical centers and scored each nodule with reference to both ACR TIRADS classification criteria and CEUS TIRADS criteria. The latter criteria were based on TIRADS in combination with information about enhancement patterns (hypo-enhancement vs hyper-enhancement) and enhancement margin (smooth vs blurred). The CEUS TIRADS criteria were adapted from prior studies [12] and incorporated our results on the application of CEUS to the evaluation of thyroid nodules (Table 1).

**Table 1.** The CEUS TIRADS diagnostic criteria

CEUS TIRADS	The CEUS diagnostic criteria
ACR TIRADS + 1 level	Hypo-enhancement and blurred enhanced margin
ACR TIRADS + 0 level	Hypo-enhancement or blurred enhanced margin
ACR TIRADS − 1 level	Iso-/hyper-enhancement and smooth enhanced margin

Abbreviations: ACR, American College of Radiology; CEUS, contrast-enhanced ultrasound; TIRADS, Thyroid Imaging Reporting and Data System.

The image dataset, alongside the associated evaluation outcomes produced by the 8 on-site senior radiologists, was sent to the analysis unit of the main medical center for additional evaluation (using the same methodology) by 2 experts (with more than 15 years of experience in thyroid US imaging). These 2 experts were blind to the outcomes produced by the 8 senior radiologists.

### Evaluation of Sonographic Features

We recorded sonographic features from conventional US images in accordance with guidelines produced by the ACR 2017 [2], the Kwak Thyroid Imaging Reporting and Data System [13], the Korean Society of Thyroid Radiology (KSThR2020) [14], the American Thyroid Association 2015 [15], and the EFSUMB [11]. The features were several thyroid nodules (single or multiple), their position (isthmus, left lobe, or right lobe), taller-than-wide shape (yes or no), morphology (regular or irregular), margin (smooth or blurred), internal echogenicity (hyper-echo, iso-echo, heterogeneous-echo, hypo-echo, or marked hypo-echo), calcification (no calcification, macrocalcification, or microcalcification), acoustic halo (uneven thickness, uniform or no halo), posterior echo (attenuation, enhancement, or no change), extra-thyroid invasion (yes or no), and blood flow (no flow, internal flow, peripheral flow, or internal and peripheral flow) within each nodule. For CEUS, we recorded enhancement intensity (hyper-enhancement, iso-enhancement, or hypo-enhancement), enhancement uniformity (heterogeneous or homogeneous), enhancement margin (smooth or blurred), ring enhancement (hyper-enhancement ring, hypo-enhancement ring), and wash-out (yes or no).

### Reference Standard

The interval between image acquisition and final confirmation of pathology (via fine needle aspiration [FNA], core needle biopsy [CNB], or surgery) did not exceed 1 month, during which there was no clinical intervention. We regarded the following as reference standards: (a) histopathologic findings from CNB according to the Korean proposal [16]; (b) cytological findings from FNA according to the Bethesda system; all nodules were confirmed from surgical resection.

### Statistical Analysis

#### Diagnostic criteria based on statistical models

To establish diagnostic criteria based on statistical models, we applied univariate analysis and multivariable logistic regression analysis to the clinical and sonographic features that were used to assess the association between malignant nodules and risk factors. We assessed the potential relation between clinical and sonographic features indicative of malignancy using Pearson correlation coefficients for all types of variables involved (whether rank, continuous, or other). For continuous variables, we performed comparisons between benign and malignant thyroid nodules using Student *t* tests or Mann-Whitney U tests. For categorical variables, we used either χ^2^ or Fisher exact tests. When applicable, model selection was performed using the forward stepwise approach. We assessed the goodness-of-fit of the multivariable logistic regression models using the Hosmer-Lemeshow test.

#### Diagnostic criteria identified by investigators

Investigators relied on ACR TIRADS to formulate diagnostic criteria for conventional US. The ACR TIRADS classification categories (1, 2, 3, 4, and 5) indicate the probability of a thyroid nodule being malignant. Each nodule identified by conventional US was assigned a TIRADS score by the radiologists.

On the basis of prior reports [6, 17, 18] and results from our analysis, we established diagnostic features for CEUS-based nodule characterization based on the enhancement pattern and margin in the nodule region. We formulated the CEUS TIRADS diagnostic criteria (Table 1) using the following procedure. If the CEUS criteria indicated a benign enhancement pattern (iso-enhancement, hyper-enhancement, and smooth enhancement margin), we subtracted 1 level from the TIRADS classification score, unless the nodule was originally assigned a TIRADS score of 2 or less. If the CEUS criteria suggested a malignant enhancement pattern (hypo-enhancement and blurred margin), we added 1 level to the TIRADS classification score. If the diagnosis based on the CEUS criteria was uncertain (hypo-enhancement or blurred margin), the TIRADS classification was left unmodified.

We generated receiver operating characteristic (ROC) curves to evaluate the diagnostic performance of the criteria developed by investigators with respect to ACR TIRADS and CEUS TIRADS. For each ROC curve, we measured the area under the curve (AUC). Pathological results were used as the reference standard. The optimal cutoff values for the prediction of malignant thyroid nodules were identified using the highest Youden index and were selected to maximize sensitivity and specificity. The Delong test was used to compare AUC values using different criteria.

We used kappa coefficients to assess interobserver consistency between senior radiologists and experts in scoring diagnostic criteria developed by investigators.

The statistical power associated with our sample size was adequate for our intended measurements. To justify this conclusion, we consider the following target values: sensitivity and specificity of combined CEUS around 80%, significance level of .05, degree of certainty (power) of 0.90, and prevalence rate of 0.50. With a target loss rate of 10%, these objectives translate into a minimum of 165 participants for inclusion in this study. Our sample size exceeded this target value.

We set our threshold for statistical significance at *P* < .05. We performed all analyses with version 19 of MedCalc and version 3.6.1 of the R software (http://www.r-project.org). We used PASS.15 to estimate the sample size.

## Results

### Demographic Characteristics

Participants were enrolled from 8 university teaching hospitals throughout China. We assigned 371 participants (249 malignant and 122 benign nodules) to the derivation cohort and 144 participants (74 malignant and 70 benign nodules) to the validation cohort. For both cohorts, participants with malignant nodules were younger (*P* < .001) with (on average) smaller nodules (*P* < .001). In the derivation cohort, those patients did not present a history of neck surgery (*P* = .035) and experienced more frequent occurrences of thyroid disease (*P* < .001) compared with participants carrying benign nodules (Table 2). The FNA specimen results were as follows: of the 303 cases (derivation cohort) and 85 cases (validation cohort), 84 and 35 cases were benign, 2 and 2 cases were indeterminate, 1 and 1 cases were follicular neoplasm, 54 and 7 cases were suspicious for malignancy, 162 and 40 cases were malignant, in the derivation and validation cohort, respectively (Table 2). The CNB specimen results: of the 68 cases (derivation cohort) and 59 cases (validation cohort), 33 and 31 cases were benign, 3 and 2 cases were indeterminate, 5 and 3 cases were follicular neoplasm, 27 and 23 cases were malignant, in the derivation and validation cohort, respectively (Table 2).

**Table 2.** Clinical features of benign and malignant thyroid nodules

Clinical features	Derivation cohort		*P* value	Validation cohort		*P* value
	Benign (N = 122)	Malignant (N = 249)		Benign (N = 70)	Malignant (N = 74)	
Age, Mean ± SD	49.07 ± 12.78	43.81 ± 11.74	<.001*^a^*	48.69 ± 12.58	42.28 ± 10.80	<.001*^a^*
Mean diameter, Mean ± SD	1.67 ± 1.12	0.96 ± 0.68	<.001*^a^*	1.88 ± 1.15	0.99 ± 0.91	<.001*^a^*
Sex: No. (%)			.387			.476
Male	31 (29.5)	74 (70.5)		18 (25.7)	23 (31.1)	
Female	91 (34.2)	175 (65.8)		52 (74.3)	51 (68.9)	
Concomitant disease: No. (%)			.886			.836
No	91 (33.1)	184 (66.9)		50 (71.4)	54 (73.0)	
Yes	31 (32.3)	65 (67.7)		20 (28.6)	20 (27.0)	
History of neck surgery: No. (%)			.035*^a^*			.235
No	119 (32.3)	249 (67.7)		68 (97.14)	74 (100.0)	
Yes	3 (100.0)	0		2 (2.86)	0 (0.0)	
History of neck cancer: No. (%)			.329			NA
No	121 (32.7)	249 (67.3)		70 (100.0)	74 (100.0)	
Yes	1 (100.0)	0		0	0	
Thyroid disease: No. (%)			<.001*^a^*			.117
No	109 (37.8)	179 (62.2)		69 (98.6)	68 (91.9)	
Yes	13 (15.7)	70 (84.3)		1 (1.4)	6 (8.1)	
BMI, Mean ± SD	24.6 ± 4.71	24.2 ± 4.65	.433	24.24 ± 3.23	24.00 ± 5.03	.807
FNA: No. (%)			<.001*^a^*			<.001*^a^*
II	82 (95.4)	2 (0.9)		33 (86.8)	2 (4.3)	
III	1 (1.2)	1 (.5)		2 (5.3)	0 (0.0)	
IV	1 (1.2)	0 (0.0)		1 (2.6)	0 (0.0)	
V	2 (2.4)	52 (24.0)		2 (5.2)	5 (10.6)	
VI	0 (0.0)	162 (74.7)		0 (0.0)	40 (85.1)	
CNB: No. (%)			<.001*^a^*			<.001*^a^*
II	32 (88.9)	1 (3.1)		30 (93.8)	1 (3.7)	
III	2 (5.6)	1 (3.1)		1 (3.1)	1 (3.7)	
IV	2 (5.6)	3 (9.4)		1 (3.1)	2 (7.4)	
VI	0 (0.0)	27 (84.4)		0 (0.0)	23 (85.2)	

Abbreviations: BMI, body mass index; CNB, core needle biopsy; FNA, fine needle aspiration.

^a^ *P* indicates a statistically significant difference.

### Diagnostic Performance for Criteria Based on Statistical Models

**Figure 2.**
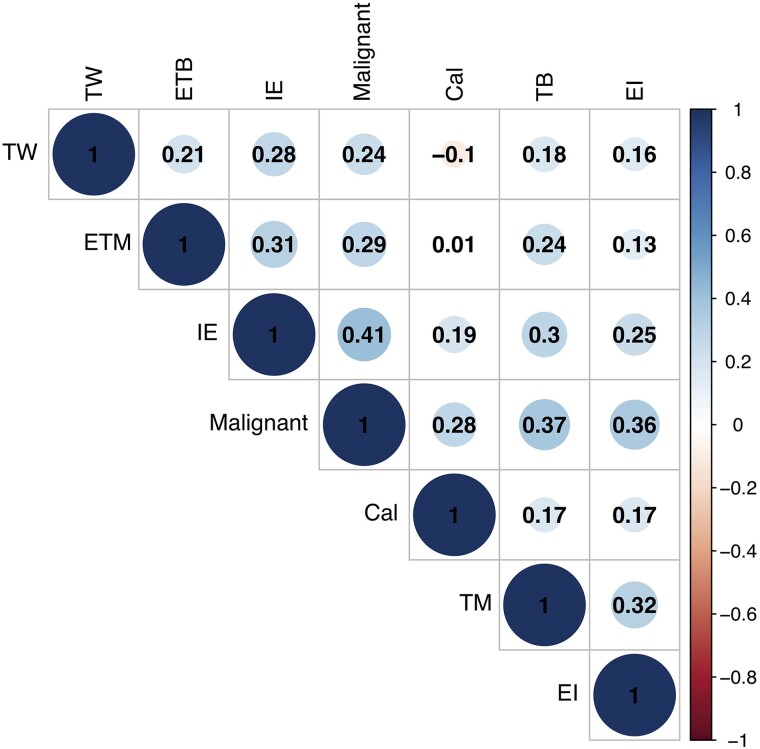
Univariate correlation matrix for US and CEUS features associated with malignant thyroid nodules. Abbreviations: Cal, calcification; TM, thyroid margin; TW, taller-than-wide; IE, internal echo; EI, enhancement intensity; ETM, enhancement margin.

Table 3 details a univariate analysis of US and CEUS sonographic features for discriminating malignant from benign thyroid nodules. With the aid of the multivariate logistic regression model, we selected the following sonographic features as part of the diagnostic criteria derived from statistical models (Table 4): taller-than-wide shape, margin, internal echo, and calcifications for US-based features; enhancement intensity and enhancement margin for CEUS-based features. We found statistically significant differences in odd ratios and high correlation for all these features (Fig. 2). On the basis of the results of multivariate analysis and correlation analysis, we selected the above US and CEUS features for inclusion in the regression model.

**Table 3.** Univariate analysis of US and CEUS sonographic imaging features for benign and malignant thyroid nodules in the derivation cohort

US imaging features	Benign (N = 122)	Malignant (N = 249)	*P* value
Number: No. (%)			.538
Single	54 (32.9)	110 (67.1)	
Multiple	68 (32.9)	139 (67.1)	
Position: No. (%)			.353
Isthmus	3 (20)	12 (80)	
Left lobe	64 (35.8)	115 (64.2)	
Right lobe	55 (31.1)	122 (68.9)	
Taller-than-wide shape: No. (%)			<.001*^a^*
No	88 (43.3)	115 (56.7)	
Yes	34 (20.2)	134 (79.8)	
Morphology: No. (%)			<.001*^a^*
Regular	57 (51.8)	53 (48.2)	
Irregular	65 (24.9)	196 (75.1)	
Margin: No. (%)			<.001*^a^*
Smooth	58 (63.7)	33 (36.3)	
Blurred	64 (31.4)	216 (68.6)	
Internal echo: No. (%)			<.001*^a^*
Hyper-echo	2 (40)	3 (60)	
Iso-echo	12 (66.7)	6 (33.3)	
Heterogeneous-echo	39 (76.5)	12 (23.5)	
Hypo-echo	65 (23.9)	207 (76.1)	
Marked hypo-echo	4 (16)	21 (84)	
Calcification: No. (%)			<.001*^a^*
No calcification	75 (46.6)	86 (53.4)	
Macrocalcification	18 (37.5)	30 (62.5)	
Microcalcification	29 (17.9)	133 (82.1)	
Acoustic halo: No. (%)			.005*^a^*
Uneven thickness	8 (18.6)	35 (81.4)	
Uniform	6 (75)	2 (25)	
No	108 (33.8)	212 (66.3)	
Posterior echo: No. (%)			.213
Attenuation	15 (28.3)	38 (71.7)	
Enhancement	7 (53.8)	6 (46.2)	
No change	100 (32.8)	205 (67.2)	
Extra-thyroid invasion: No. (%)			.213
Yes	6 (13.6)	38 (86.4)	
No	116 (35.5)	211 (64.5)	
Blood flow: No. (%)			.007*^a^*
No flow	25 (27.5)	66 (72.5)	
Internal flow	36 (40.9)	52 (59.1)	
Peripheral flow	9 (16.4)	46 (83.6)	
Internal and peripheral flow	52 (38)	85 (62)	
ACR TIRADS: No. (%)			<.001
1	30 (100)	0	
2	35 (76.1)	11 (23.9)	
3	21 (47.7)	23 (54.3)	
4	32 (16.1)	167 (83.9)	
5	4 (7.7)	48 (92.3)	
**CEUS imaging features**			
Enhancement intensity: No. (%)			<.001*^a^*
Hyper-enhancement	15 (75)	5 (25)	
Iso-enhancement	71 (48.3)	76 (51.7)	
Hypo-enhancement	36 (17.6)	168 (82.4)	
Enhancement uniformity: No. (%)			.001*^a^*
Heterogeneous	57 (26.1)	161 (73.9)	
Homogeneous	65 (42.5)	88 (57.5)	
Enhancement margin: No. (%)			<.001*^a^*
Smooth	41 (62.1)	25 (37.9)	
Blurred	81 (26.6)	224 (73.4)	
Ring enhancement: No. (%)			.001*^a^*
Hyper-enhancement ring	28 (56)	22 (44)	
Hypo-enhancement ring	14 (36.8)	24 (63.2)	
No	80 (28.3)	203 (71.7)	
Wash-out: No. (%)			.322
Yes	22 (28.2)	56 (71.8)	
No	100 (34.1)	193 (65.9)	

Abbreviations: ACR, American College of Radiology; CEUS, contrast-enhanced ultrasound; TIRADS, Thyroid Imaging Reporting and Data System; US, ultrasound.

^*a*^Indicates statistically significant *P* values.

**Table 4.** Results from multivariate logistic regression models applied to US and CEUS sonographic features for differentiating between benign and malignant thyroid nodules in the derivation cohort

Modality	Level	OR	95% CI	*P* value
**US features**				
Taller-than-wide shape	Yes	2.16	1.16, 4.00	.015*^a^*
Margin	Blurred	2.34	1.23, 4.44	.0097*^a^*
Calcification	Microcalcification	2.97	1.62, 5.44	.0004*^a^*
Internal echo	Marked hypo-echogenicity/ Hypo-echogenicity	2.94	1.38, 6.24	.005*^a^*
**CEUS features**				
Enhancement intensity	Hypo-enhancement	2.87	1.62, 5.09	.0003*^a^*
Enhancement margin	Blurred	3.10	1.48, 6.50	.0028*^a^*

Abbreviations: CEUS, contrast-enhanced ultrasound; OR, odds ratio; US, ultrasound.

^*a*^Indicates statistically significant *P* values.

**Figure 3.**
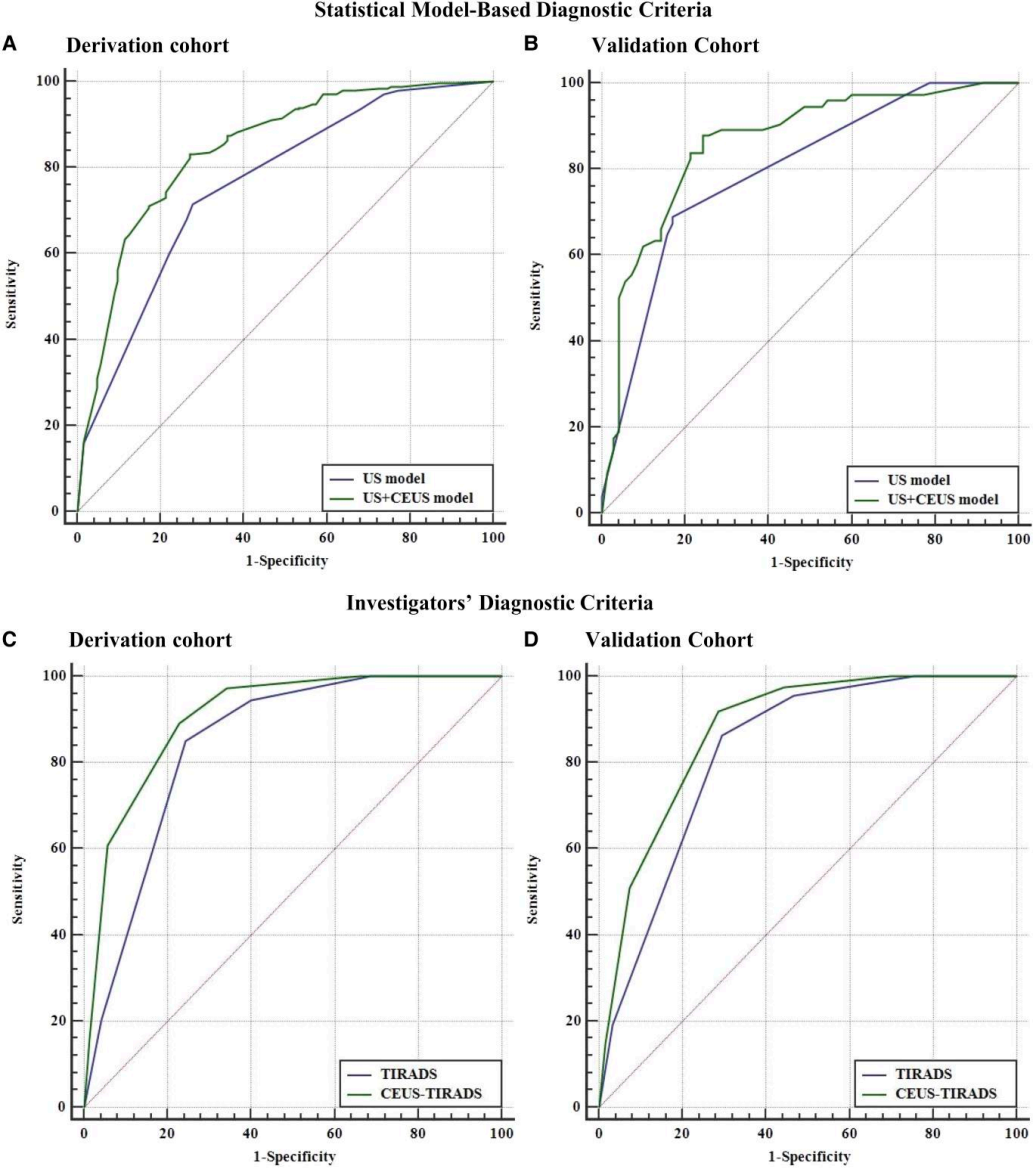
The discrimination power of US and US + CEUS models was assessed via receiver operating characteristic curves in the derivation cohort (A) and validation cohort (B). The discrimination power of ACR TIRADS (US) and CEUS + TIRADS criteria was assessed via receiver operating characteristic curves in the derivation cohort (C) and validation cohort (D). Abbreviations: ACR, American College of Radiology; CEUS, contrast-enhanced ultrasound; TIRADS, Thyroid Imaging Reporting and Data System; US, ultrasound.

When applied to the derivation cohort, the AUC value for the US + CEUS model (0.84; 95% CI, 0.80–0.88) was higher than the corresponding value for the US model (0.76; 95% CI, 0.72–0.81; *P* = .001; Fig. 3A). This result was confirmed with reference to the validation cohort: again, the US + CEUS model produced a significantly higher AUC value (0.86; 95% CI, 0.80–0.91) than the US model (0.80; 95% CI, 0.72–0.86; *P* = .036; Fig. 3B). The Akaike information criterion and the improvement in integrated discrimination provide evidence that the model based on US + CEUS features outperformed the US model when discriminating between malignant and benign thyroid nodules (Table 5).

**Table 5.** Comparison of diagnostic performance for criteria from statistical models based on US and US + CEUS

Diagnostic criteria	AIC	Cutoff	AUC (95% CI)	Sensitivity (95% CI)	Specificity (95% CI)	AUC difference	*P*_AUC_ value	IDI difference	*P*_IDI_ value
**Derivation cohort**									
US model	366.4	0.514	0.76 (0.72-0.81)	71.5% (65.4-77%)	72.1% (63.3%-79.9%)	0.08	.001*^a^*	0.05	<.001*^a^*
US + CEUS model	347.7	0.610	0.84 (0.80-0.88)	83.1% (77.9%-87.6%)	72.9% (64.2%-80.6%)				
**Validation cohort**									
US model	163.5	0.514	0.80 (0.72-0.86)	68.9% (57.1%-79.2%)	82.9% (72.0%-90.8%)	0.06	.036*^a^*	0.23	<.001*^a^*
US + CEUS model	140.5	0.543	0.86 (0.80-0.91)	87.8% (78.2%-94.3%)	75.7% (64.0%-85.2%)				

*P*_AUC_ value: Comparison of the area under the curve between US model and US + CEUS model.

*P*_IDI_ value: Comparison of the improvement in integrated discrimination between US model and US + CEUS model.

Abbreviations: AIC, Akaike information criterion; AUC, area under the curve; CEUS, contrast-enhanced ultrasound; IDI, integrated discrimination improvement; US, ultrasound.

^*a*^Indicates statistically significant *P* values.

### Diagnostic Performance of Criteria Identified by Investigators

In accordance with the criteria formulated by investigators (Table 6), we measured a significant difference in AUC values between ACR TIRADS (0.83; 95% CI, 0.78–0.86) and CEUS TIRADS (0.87; 95% CI, 0.84–0.91; *P* < .001) with reference to the derivation cohort (Fig. 3C). This result was confirmed with reference to the validation cohort: the AUC value for CEUS TIRADS (0.91; 95% CI, 0.85–0.95) was again higher than the AUC value for ACR TIRADS (0.84; 95% CI, 0.77–0.90; *P* < .001; Fig. 3D).

**Table 6.** Comparison of diagnostic performance for criteria formulated by investigators based on ACR TIRADS and CEUS TIRADS

Diagnostic criteria	Cutoff	AUC (95% CI)	Sensitivity (95% CI)	Specificity (95% CI)	AUC difference	*P*_AUC_ value
**Derivation cohort**						
ACR TIRADS	3	0.83 (0.78-0.86)	86.4% (81.4%-90.4%)	70.5% (61.6%-78.4%)	0.04	<.001*^a^*
CEUS TIRADS	3	0.87 (0.84-0.91)	91.9% (87.9%-95.0%)	72.9% (64.2%-80.6%)		
**Validation cohort**						
ACR TIRADS	3	0.84 (0.77-0.90)	85.1% (75.0%-92.3%)	75.7% (64.0%-85.2%)	0.07	<.001*^a^*
CEUS TIRADS	3	0.91 (0.85-0.95)	89.2% (79.8%-95.2%)	77.1% (65.6%-86.3%)		

*P*_AUC_ value: Comparison of the area under the curve between ACR TIRADS and CEUS TIRADS.

Abbreviations: ACR, American College of Radiology; AUC, area under the curve; CEUS, contrast-enhanced ultrasound; TIRADS, Thyroid Imaging Reporting and Data System.

^*a*^Indicates statistically significant *P* values.

### Participants With Special Benefits From CEUS

**Figure 4.**
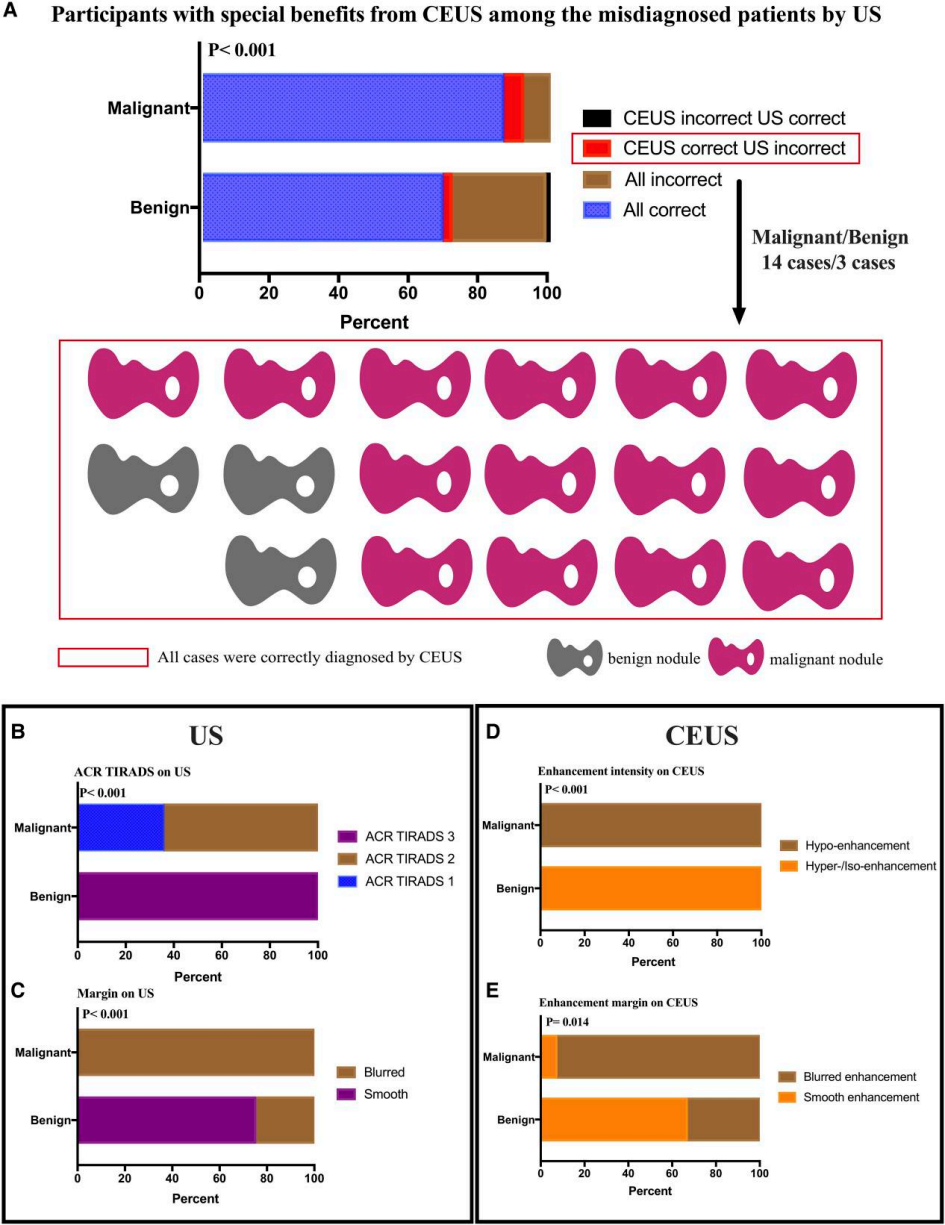
Participants with special benefits from CEUS. Final diagnostic results from ACR TIRADS and CEUS TIRADS (A). Seventeen participants with benefits from CEUS were misdiagnosed by ACR TIRADS (B). Margin is the only US feature that helps in the diagnosis of these patients (C). Enhancement intensity (D) and enhancement margin (E) on CEUS can help in the diagnosis of these patients.

On the basis of the final results from ACR TIRADS and CEUS TIRADS, these criteria supported correct diagnosis for 299 cases (84 benign and 215 malignant). Of the misdiagnosed participants, 17 cases (3 benign and 14 malignant) were correctly diagnosed only by CEUS TIRADS (Fig. 4A). In these participants, 3 benign nodules were evaluated as TIRADS-3, 5 malignant nodules as TIRADS-1, and 9 malignant nodules as TIRADS-2 (Fig. 4B). Three benign nodules and one malignant nodule were evaluated as smooth margin by US. Thirteen malignant nodules were evaluated as blurred margin by US (*P* < .001; Fig. 4C). On the basis of CEUS, 3 benign nodules were evaluated as hyper-enhancement or iso-enhancement, and 14 malignant nodules were evaluated as hypo-enhancement (*P* < .001; Fig. 4D). In addition, 2 benign nodules and 1 malignant nodule were evaluated as smooth enhancement margin. One benign nodule and 13 malignant nodules were evaluated as blurred enhancement margin (please refer to Fig. 4E).

### Interobserver Agreement

We found good interobserver agreement between on-site radiologists and experienced radiologists with respect to both ACR TIRADS and enhancement patterns (k = 0.776 and k = 0.808, respectively).

## Discussion

**Figure 5.**
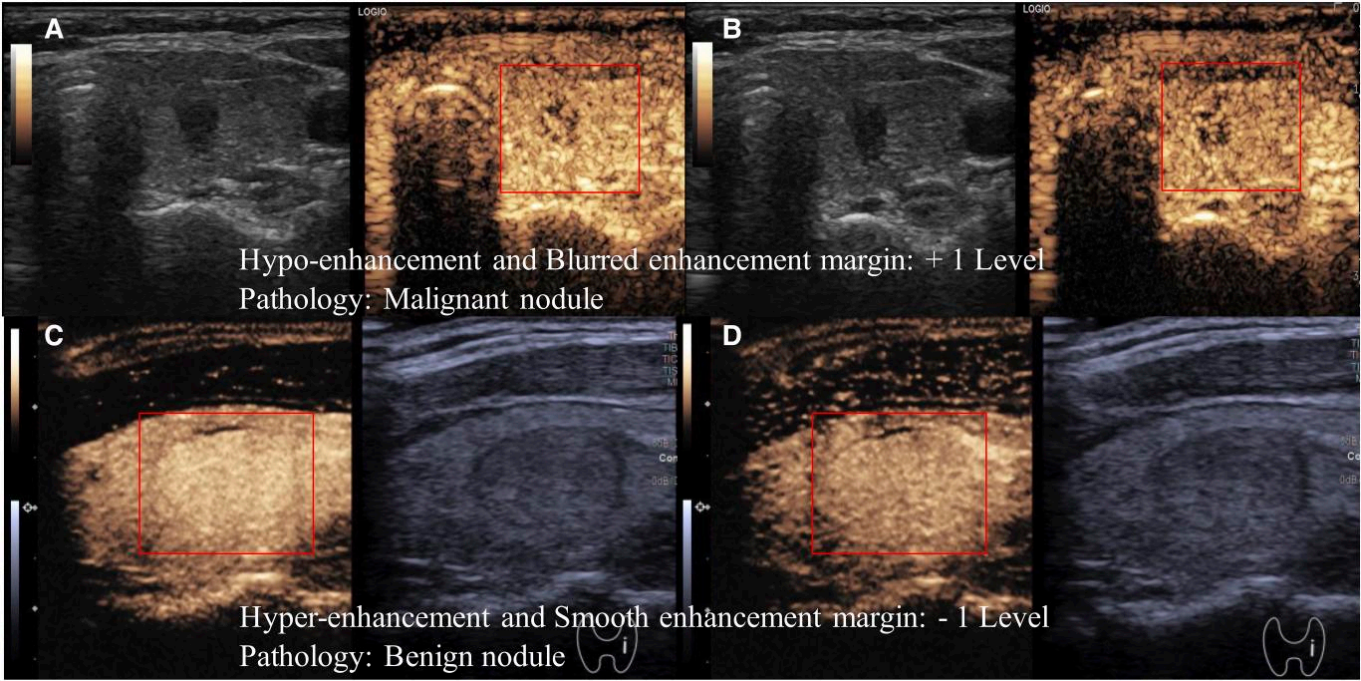
Hypo-enhancement and blurred enhancement margin (A, B) are shown for a malignant thyroid nodule. Hyper-enhancement and smooth enhancement margin (C, D) are shown for a benign thyroid nodule at 15 seconds and 30 seconds, respectively.

CEUS is an imaging modality that is increasingly being used for thyroid nodule diagnosis. To our knowledge, this is the first prospective multicenter study that has evaluated the clinical value of CEUS in thyroid (Fig. 5). We explored its diagnostic performance in the case of thyroid nodules by comparing US alone with a combination of both US and CEUS. Notably, our results indicate that CEUS may improve diagnostic accuracy when applied in combination with conventional US, and this may therefore carry implications for future revisions of the EFSUMB guidelines which, at present, do not recommend CEUS for clinical use.

The ACR TIRADS scheme proposed by the American College of Radiology emphasizes the value of sonographic features and serves as a standardized system for differentiating between benign and malignant thyroid nodules. Its recommendations were agreed upon by wide consensus [19]. In addition to relying on this established system, our study produced consistent results from 2 comparative analytical strategies. On the basis of diagnostic criteria developed through statistical models, we found that the combined strategy (US + CEUS) produced a significantly higher AUC value (0.84) than that obtained from US alone (0.76, *P* < .001). The method based on US + CEUS features produced higher AUC values for the derivation (0.84) and validation cohorts (0.86), alongside improvements in integrated discrimination. Although at the optimal cutoff values using the highest Youden index, the specificity of the CEUS + US model is slightly lower than that of the US model, the sensitivity and AUC of the CEUS + US model are significantly higher than that of the US model in the validation cohort. When the analysis was based on diagnostic criteria developed by investigators, AUC values were significantly different between ACR TIRADS and CEUS TIRADS with reference to both cohorts.

Our baseline analysis applied to the derivation cohort shows that prior occurrence of thyroid disease was a risk factor for thyroid cancer. Several studies have proposed that Hashimoto thyroiditis or nodular goiter may be related to potential malignant transformations [20]. The multivariate logistic regression model based on sonographic features from US and CEUS methodologies identified several significant risk factors for the diagnosis of malignant thyroid nodules, such as taller-than-wide shape, margin, internal echo, and calcifications visible under US imaging. Hypo-enhancement and an unclear enhancement margin were significantly associated with malignant nodules. In our study, all malignant nodules were identified as papillary thyroid cancers. The high predominance of papillary structures can explain the enhancement features of papillary thyroid cancers throughout the tumor on microscopy: according to the classification criteria developed by the World Health Organization, papillary thyroid cancer always presents as an invasive neoplasm with poorly defined morphologies on macroscopic evaluation [21]. Hypo-enhancement is the most frequent predictor of malignancy on CEUS, with high sensitivity, specificity, and accuracy values of 77.3%, 93.9%, and 90.0%, respectively [6]. The blurred enhancement margin observed on CEUS was similar to the blurred margin on the US, although this feature may be magnified on CEUS. Other enhancement features, such as ring enhancement, were predictive of benign lesions, while heterogeneous enhancement helped detect malignant lesions, as reported by Zhang et al [17].

Our final results confirmed that 17 cases (3 benign and 14 malignant) were incorrectly diagnosed by TIRADS, but correctly diagnosed by CEUS TIRADS. In particular, enhancement intensity and enhancement margin from CEUS provide auxiliary diagnostic value. Our findings suggest that the diagnostic value of CEUS in combination with US was higher than conferred by US alone. This result is broadly consistent with prior studies [6, 7, 10]. For example, Huang et al [18] reported a significantly higher AUC value for CEUS (0.835) compared with TIRADS (0.738). At the same time, other studies (such as [7]) did not report any obvious improvement from using CEUS in place of US. We attribute the improved reliability and resolving power of our results to the adoption of a prospective design and the large sample used in this study. Therefore, CEUS examinations could justify the appropriate use of contrast media in combination with US and clinical information.

Our study had major limitations. Our sample contains a larger number of malignant than benign nodules. This inherent selection bias was likely inevitable because our participants presented suspicious nodules on US that warranted biopsy or surgery, and these procedures are typically performed on nodules that are suspicious for malignancy. For patients with surgery for benign nodules, the main reasons were that there were compressive symptoms or that the results of the FNA nodule biopsy and CNB were indeterminate and the patient was anxious so ultimately opted for surgical resection of the thyroid nodule.

In conclusion, on the basis of diagnostic criteria formulated by investigators and on those identified by statistical models, we found evidence that contrast-enhanced ultrasound could provide complementary information to conventional US for the diagnosis of benign and malignant nodules. This finding should prompt radiologists to combine CEUS with ultrasound for effective diagnosis of thyroid nodules.

## Data Availability

Some or all datasets generated during and/or analyzed during the current study are not publicly available but are available from the corresponding author on reasonable request.

## Abbreviations

ACR: American College of Radiology
AUC: area under the curve
CEUS: contrast-enhanced ultrasound
CNB: core needle biopsy
EFSUMB: European Federation of Societies for Ultrasound in Medicine and Biology
FNA: fine needle aspiration
ROC: receiver operating characteristic
TIRADS: Thyroid Imaging Reporting and Data System
US: ultrasound
